# Editorial: The Fungal Cell Wall

**DOI:** 10.3389/fmicb.2020.01682

**Published:** 2020-09-09

**Authors:** Fausto Almeida, Joshua D. Nosanchuk, Gustavo Alexis Niño-Vega

**Affiliations:** ^1^Department of Biochemistry and Immunology, Ribeirão Preto Medical School, University of São Paulo, São Paulo, Brazil; ^2^Division of Infectious Diseases, Department of Medicine, Albert Einstein College of Medicine, New York, NY, United States; ^3^Department of Microbiology, Albert Einstein College of Medicine, New York, NY, United States; ^4^Division de Ciencias Naturales y Exactas, Departamento de Biologia, Universidad de Guanajuato, Guanajuato, Mexico

**Keywords:** cell wall, fungi, pathogenic fungi, pathogenic fungal disease, non-pathogenic fungi, fungal structure, fungal metabolism

A robust understanding of the complexity and functionality of the fungal cell wall is crucial to the development of new therapeutic and prophylactic strategies. The cell wall plays several key functions in fungal pathobiology as diverse factors, such as cell shape, encapsulation, and rigidity, influence events during interaction with the host (Gow et al., 2017). These interactions may be proinflammatory or may subvert host responses. The fungal cell wall has a flexible structure that is highly complex and intricately organized of α- and β- linked glucans, chitin, glycoproteins, and pigments (Gow et al., 2017).

The researchers who contributed to this Research Topic presented 13 themed articles that highlighted the latest advances in our understanding of the biological importance of the fungal cell wall. For example, Garcia-Rubio et al. summarized recent findings on the characteristics and influence of cell wall components on fungi-host interaction with a specific focus on three fungal species, *Aspergillus fumigatus, Candida albicans*, and *Cryptococcus neoformans*. Lima et al. discussed the wealth of information available regarding antifungal therapy and the development of antimicrobial resistance subsequent to cell wall modifications by several important human pathogenic fungi. Patel and Free addressed the genetics and biochemistry that lead to the formation of the complex *Neurospora crassa* cell wall, comparing this species' cell wall with that of other fungal species.

Two reports focused on the dermatophyte *Trichophyton rubrum*. Martins et al. evaluated RNA-seq results under stress conditions, using undecanoic acid and acriflavine as well as the influence of the carbon source, on the modulation of genes regulating *T. rubrum* cell wall metabolism. These investigators described that keratin mimics the host environment and undecanoic acid and acriflavine present non-specific antifungal activity against *T. rubrum*. Thus, the authors identified genes putatively encoding *T. rubrum* virulence factors. Celestrino et al. verified that Toll-like receptor 2 (TLR2) is required for efficient phagocytosis of *T. rubrum* conidia by adherent monocytes, and the absence of TLR2 signaling in human monocytes impairs the expected inflammatory responses.

In the work by Sun et al., we learnt that the farnesyltranferase β subunit Ram1 regulates pathogenicity, mating, and cell wall integrity, and it also plays an important role as a virulence factor in the sugarcane smut fungus *Sporisorium scitamineum*. Miyazawa et al. described that both α-1,3-glucan and galactosaminogalactan are adhesive molecules and these glucans contribute to aggregation on the hyphal surface of *Aspergillus oryzae*. In *P. brasiliensis*, Souza et al. verified that cell wall α-glucan induced differentiation of dendritic cells, which could contribute to pathogen persistence since this process potentially affects Th1 polarization. de Oliveira et al. demonstrated that thioredoxin reductase 1 is a highly immunogenic surface antigen in the cell walls of *Candida albicans, Paracoccidioides* spp., and *Cryptococcus neoformans*, and that the enzyme has conserved epitopes in fungi, but there are no homologs in humans. In *Candida parapsilosis*, Oh et al. verified the nature of the agglutinin-like sequence gene family, which encodes cell-surface glycoproteins involved in the adhesion of fungal cells to host and abiotic surfaces. The authors also demonstrated allelic variability and expression patterns. The Zheng et al. verified that the deletion of a gene responsible for coding a calcineurin homolog from *Talaromyces marneffei* affects germination, cell wall integrity, morphogenesis, and resistance to external stresses. Orner et al. demonstrated that the cell-wall-associated antiphagocytic protein 1 and laccase enzymes (named Lac1 and Lac2) play important roles in increasing resistance to amphotericin B and host-mediated killing during infection as well as enhancing the subsequent accumulation of old *C. neoformans* cells (10 generations old), which melanized to a greater extent than younger *C. neoformans* cells (0–2 generations old). Helal et al., which includes one of us, presented the first description that pan-antigens displayed on the cell surface of pathogenic fungi can effectively be targeted with radioimmunotherapy. The authors described the ability of a radiolabeled anti-(1-3)-β-D-glucan antibody to specifically target *Blastomyces dermatitidis in vitro* and *in vivo*. Furthermore, this specific radioimmunotherapy selectively killed *B. dermatitidis* under both *in vitro* and *in vivo* conditions.

In conclusion, this themed collection enhances our knowledge of the diverse functions of the fungal cell wall in host-pathogen interactions, and the papers particularly highlighted potential targets and methods for antifungal development, reinforcing the relevance of studies focused on elucidating the biology of the fungal cell wall.

## Author Contributions

FA drafted the Editorial while JN and GN-V contributed to editing. All authors conceived and designed the work and provided final approval of the version to be published.

## Conflict of Interest

The authors declare that the research was conducted in the absence of any commercial or financial relationships that could be construed as a potential conflict of interest. The handling editor declared a shared affiliation with one of the authors, FA, at time of review.
